# Hierarchical Porous Carbon—PLLA and PLGA Hybrid Nanoparticles for Intranasal Delivery of Galantamine for Alzheimer’s Disease Therapy

**DOI:** 10.3390/pharmaceutics12030227

**Published:** 2020-03-04

**Authors:** Stavroula G. Nanaki, Konstantinos Spyrou, Chryssa Bekiari, Pelagia Veneti, Turki N. Baroud, Niki Karouta, Ioannis Grivas, Georgios C. Papadopoulos, Dimitrios Gournis, Dimitrios N. Bikiaris

**Affiliations:** 1 Laboratory of Polymers and Colors Chemistry and Technology, Department of Chemistry, Aristotle University of Thessaloniki, 54124 Thessaloniki, Greece; sgnanaki@chem.auth.gr (S.G.N.); pelagia1990@gmail.com (P.V.); 2 Department of Materials Science and Engineering, University of Ioannina, 45110 Ioannina, Greece; konstantinos.spyrou1@gmail.com (K.S.); nikhkarouta@gmail.com (N.K.); dgourni@uoi.gr (D.G.); 3 Laboratoryof Anatomy, Histology and Embryology, School of Veterinary Medicine, Aristotle University of Thessaloniki, 54124 Thessaloniki, Greece; chmpekia@vet.auth.gr (C.B.); janos@vet.auth.gr (I.G.); gpapadop@vet.auth.gr (G.C.P.); 4 Department of Mechanical Engineering, King Fahd University of Petroleum & Minerals, Dhahran 31261, Saudi Arabia; turkibaroud@kfupm.edu.sa

**Keywords:** galantamine, Alzheimer’s, poly(l-lactic acid), poly(lactide-*co*-glycolide), hierarchical porous carbon, nanoencapsulation, drug delivery, intranasal delivery

## Abstract

In the present study, poly(l-lactic acid) (PLLA) and poly(lactide-*co*-glycolide) (PLGA) hybrid nanoparticles were developed for intranasal delivery of galantamine, a drug used in severe to moderate cases of Alzheimer’s disease. Galantamine (GAL) was adsorbed first in hierarchical porous carbon (HPC). Formulations were characterized by FT-IR, which showed hydrogen bond formation between GAL and HPC. Furthermore, GAL became amorphous after adsorption, as confirmed by XRD and differential scanning calorimetry (DSC) studies. GAL was quantified to be 21.5% *w*/*w* by TGA study. Adsorbed GAL was nanoencapsulated in PLLA and PLGA, and prepared nanoparticles were characterized by several techniques. Their sizes varied between 182 and 394 nm, with an exception that was observed in nanoparticles that were prepared by PLLA and adsorbed GAL that was found to be 1302 nm in size. DSC thermographs showed that GAL was present in its crystalline state in nanoparticles before its adsorption to HPC, while it remained in its amorphous phase after its adsorption in the prepared nanoparticles. It was found that the polymers controlled the release of GAL both when it was encapsulated alone and when it was adsorbed on HPC. Lastly, PLGA hybrid nanoparticles were intranasally-administered in healthy, adult, male Wistar rats. Administration led to successful delivery to the hippocampus, the brain area that is primarily and severely harmed in Alzheimer’s disease, just a few hours after a single dose.

## 1. Introduction

Alzheimer’s disease (AD) is one of the major causes of senile dementia and is characterized by progressive neurodegeneration that causes gradual neuronal and memory loss, as well as cognition impairment [1]. The ever-growing prevalence of AD and its detrimental effect on patients’ quality of life has encouraged the scientific community to thoroughly search for effective treatments. Despite all activity, however, the only approved treatment modalities for AD are acetylcholinesterase (AChE) inhibitors and memantine, which merely serve as symptomatic cognitive-enhancing drugs and cannot alter the course of the disease [2].

Galantamine (GAL) a reversible and competitive AChE inhibitor that is used for the treatment of mild-to-moderate dementia of the AD type. It acts by inhibiting AChE, increasing the concentration and thereby the activity of acetylcholine in certain parts of the brain. Hippocampal formation is the brain region that is responsible for learning and memory, and it is this region that is primarily and severely harmed in AD, leading to the cognitive decline of AD patients. Chronic GAL treatment has been shown to significantly lower Aβ amyloid plaque density in the hippocampus and to improve cognitive and behavioral symptoms in a mouse model of AD disease. The efficacy of GAL has also been reported in patients with severe Alzheimer’s disease [3]. Approved formulations that are associated with the oral administration of the drug come in solution, capsules, or film-coated tablets [4]. The oral administration of GAL in AD patients, however, is accompanied by severe gastrointestinal side effects, such as nausea, vomiting, and diarrhea, that may lead to the suspension of the treatment [5]. Furthermore, oral administration, in addition to the drawbacks mentioned above, is difficult for people suffering from AD due to their intellectual capacity, which can lead to noncompliance with their therapeutic regimen. Innovative administration systems are crucial and need to be developed.

There have already been published studies focusing on different routes for GAL delivery. Mufamadi et al. [6] proposed a liposome-embedded, polymeric scaffold for its intravenous delivery, showing a prolonged release of GAL for about 40 days. Ameen and Michniak-Kohn [7] developed a pressure-sensitive adhesive patch for the transdermal delivery of GAL by using limonene and oleic acid as penetration enhancers. Khatavkar et al. [8] developed novel monolithic matrix mini tablets for oral administration with enhanced properties compared to a commercial system. Carbon dots [9], a form of carbon nanoparticles, have also been used in GAL intravenous delivery.

The intranasal (IN) delivery of GAL for management of AD could be an alternative strategy to orally-administered GAL and has recently gained attention. IN administration overcomes the selective permeability of the blood–brain-barrier and the first pass metabolism in liver, leading to high drug accumulations in the brain [10,11]. The nose-to-brain drug delivery also avoids gastrointestinal and other systemic side effects that are triggered by the direct interaction of GAL molecules with cells of the intestinal mucosa [12]. However, despite the above benefits of the nose-to-brain delivery of GAL, the inability of IN administration to retain constant therapeutic levels of GAL in the brain represents a serious drawback. Just after IN delivery of GAL, the initial high short-lived pharmacokinetic peak is followed by a longer period where GAL is present in decreasing concentrations below the one that is therapeutically required (‘burst and tailing phenomenon’). To overcome this issue, GAL is usually encapsulated in nanoparticles, of various forms, that function as drug carriers and protect GAL from degradation, leading to a gradual drug release in the brain and a more efficient AChE inhibition [13].

Previous studies have reported that the IN delivery of GAL-loaded flexible liposomes in rats enhances the efficiency of AChE inhibition in the brain as compared to orally-administrated GAL [14]. GAL/chitosan complex nanoparticles were also delivered IN to adult rats and exhibited a significant decrease in AChE levels and activity as compared to an IN-administered GAL-saline solution [10]. GAL/chitosan complex nanoparticles were also capable to enter the brain within 1 h after administration and were detected in various brain regions, including the hippocampus, which is severely harmed in AD [15].

Aliphatic polyesters have already gained acceptance in pharmaceutical technology. They are extensively used in drug delivery systems due to their properties including biocompatibility, biodegradability, and the protection of the drug from decomposition after its administration. Furthermore, polymers can control the delivery of the drug in the desired time and mode, something that renders them ideal for pharmaceutical uses. Polycaprolactone (PCL), polylactide (PLA), and their copolymers and blends have already been used in various studies by our team in pharmaceutical applications [16,17,18,19,20]. Furthermore, poly(lactide-*co*-glycolide) (PLGA), with lactide:glycolide ratios of 75/25 and 50/50 *w*/*w* has already been studied in microparticle preparation with paclitaxel [21] or paliperidone [22,23], an anticancer and an antipsychotic drug, respectively, either alone or by absorbing the drugs on silica-based nanoparticles (SBA-15 and MCF). The incorporation of the drugs in these nanoparticles and their further incorporation in polymeric microparticles was found to lead to the controlled release of the drugs.

Here, we present a hybrid delivery system based on GAL adsorbed on hierarchical porous carbon (HPC) encapsulated into polyester nanoparticles. Three polymers—poly(l-lactic acid) (PLLA) and poly(lactide-*co*-glycolide) (PLGA) copolymers with 75/25 and 65/35 *w*/*w* ratios—were used for the encapsulation of GAL. We note that this is the first time that HPCs have been used in pharmaceutical formulations.

HPCs represent a new class of porous materials. They combine an interconnected network of macro-, meso- and micropores in a simple material platform [24]. They are easy to fabricate, and they possess tunable porosities, surface areas, and controlled physical and chemical properties. As a result, they have potential in various applications including catalysis, separation, sensing, energy conversion and storage, and water desalination and treatment [25,26,27,28]. In this work, we evaluate HPCs as novel carriers for drug delivery and the controlled release of GAL. This interconnected porous network provides accessibility and active sites for GAL adsorption, which leads to high loadings of GAL that are delivered to the brain along the olfactory nerves. The gradual release of the drug, therefore, combines both targeted and slow and sustained release.

The hybrid nanoparticles were fully characterized, IN administered to adult male Wistar rats, and examined for their ability to enter the brain and reach the hippocampus. This is the first study of its kind and represents a breakthrough in the field.

## 2. Materials and Methods

### 2.1. Materials

Sodium cholate (≥99%) and poly(l-lactic acid) (PLLA) (Mn = 20,000 Da and polydispersity index (PDI) ≤ 1.3) were purchased from Sigma Aldrich Chemical Co (Steinheim, Germany). Poly(lactide-*co*-glycolide) (PLGA) of the 75/25 and 65/35 *w*/*w* ratios was kindly donated by Corbion (Amsterdam, Netherlands). Galantamine was kindly donated by Pharmathen S.A. (Athens, Greece). All other chemicals that were used were of analytical grade. Solvents that were used in HPLC analysis were of HPLC grade.

### 2.2. Synthesis of HPC

HPC was synthesized according to a well-established procedure [25]. Briefly, sucrose (the carbon precursor) was mixed with a colloidal silica suspension (40 wt% in H_2_O, 20 nm, acting as the template) in a 2:1 silica: sucrose ratio. Then, the mixture was freeze-casted in a liquid nitrogen bath, followed by freeze-drying for 2 days at room temperature under 0.014 mbar. The solid mixture was then carbonized under a continuous flow of nitrogen gas at 1050 °C (at 180 °C/h heating rate) for 3 h. The silica template was etched out by mixing the solid mixture with 3 M sodium hydroxide at 80 °C for 12 h. The resulting material was washed several times by filtration in order to remove the NaOH, as indicated by bringing the pH to around 7. Finally, in order to generate micropores and/or widen the existing small mesopores, the carbon powder was activated under the flow of CO_2_ gas at 950 °C for 8 h. A graphical representation for the synthetic procedure was reported in [27].

### 2.3. Galantamine’s Adsorption to HPC

One-hundred milligrams of galantamine was dissolved in 100 mL of methanol. Fifteen milligrams of HPC were added to the solution, and the resulting dispersion was left under magnetic stirring for 24 h. HPC with adsorbed galantamine was isolated by centrifugation at 12,500 rpm for 20 min. The precipitate was collected, washed with water once in order to remove excess galantamine, and freeze-dried. The amount of the drug adsorbed was determined by TGA.

For in vivo experiments, Rhodamine (Rhod) was also adsorbed with GAL to HPC, and the quantity of GAL was also determined by TGA before nanoparticles’ preparation.

### 2.4. Preparation of Nanoparticles

Polymeric nanoparticles containing HPC with adsorbed galantamine were prepared by a solid-oil–water (s/o/w) modified double emulsification method. According to this procedure, 50 mg of a polymer—either PLLA, PLGA 75/25, or PLGA 65/35 *w*/*w*—was dissolved in 5 mL of dichloromethane. Then, 5 mg of galantamine-adsorbed HPC was inserted into the polymeric solution and dispersed by using a probe sonicator for 1 min at cycle 1 and amplitude 100% (Germany, Hielscher Ultrasound Technology, Model UP100H). The dispersion was added to 20 mL of a sodium cholate aqueous solution, 0.1% *w*/*v* in concentration, and sonicated for 1 min. After that, it was left under magnetic stirring until all dichloromethane was evaporated. The obtained nanoparticles were isolated by centrifugation at 9500 rpm for 20 min and washed with water. The nanoparticles were finally freeze-dried and stored at 4 °C until further use. Nanoparticles containing neat galantamine were also prepared for comparison by following the same procedure described above (but in the absence of HPC). Nanoparticles of PLGA 65/35 with HPC–GAL–Rhod were also prepared by the same procedure for in vivo studies. The proper nanoparticles were washed twice with water in order to ensure that no quantity of Rhod was deposited to their surface.

### 2.5. Characterization

#### 2.5.1. BET and BJH Structural Characterization

Nitrogen adsorption−desorption isotherms were carried out on a Micrometrics ASAP2020 instrument at 77 K. Specific surface areas were calculated from Brunauer–Emmett–Teller (BET) model, and the mesopore volume was determined from the Barrett–Joyner–Halenda (BJH) model.

#### 2.5.2. UV–Vis

UV–Vis spectra were recorded on a Shimadzu UV-2401PC two-beam spectrophotometer (Shimadzu Scientific Instruments, Columbia, MD, USA) in the range of 200−800 nm at a step of 0.5 nm by using a combination of deuterium and halogen lamps as the light source.

#### 2.5.3. FE-SEM and SEM

The surface morphology of the prepared HPC was investigated by FE-SEM (field emission scanning electron microscopy) Tescan Mira3 FESEM (Tescan Orsay Holding, Brno-Kohoutovice, Czech Republic). Imaging was performed at a very high resolution (1 nm at 30 keV and 2 nm at 1 keV). Performance was conducted at particularly low accelerating voltages (i.e., 0.2–1 kV when using beam deceleration mode).

SEM images were obtained with a JEOL 2011 electron microscope (JEOL Ltd., Tokyo, Japan). Samples of the prepared nanoparticles were coated with carbon prior to imaging, and images were collected under the following operating conditions: accelerating voltage 20 kV, probe current 45 nA, and counting time 60 s.

#### 2.5.4. X-ray Photoelectron Spectroscopy (XPS)

To analyze the surface chemistry of the hierarchical porous carbons, X-ray photon spectroscopy XPS was carried out by using Surface Science Instruments (SSX100) (SPECS GmbH, Berlin, Germany) with monochromatic Al Kα X-rays (1486.6 eV).

#### 2.5.5. TGA

Thermogravimetric analysis (TGA) was carried out in a Setsys 16/18 TG-DTA (SETARAM Instrumentation, Caluire, France) instrument. Samples (10 ± 0.5 mg) were placed in alumina crucibles. An empty alumina pan was used as reference. Samples were heated from ambient temperature to 600 °C in a 50 mL/min flow of N_2_. A nominal heating rate of 20 °C/min was used, and continuous records of sample temperature, sample weight and heat flow were taken. All measurements were performed in triplicate.

#### 2.5.6. Fourier-Transformed Infrared Spectroscopy (FT-IR)

FT-IR spectra were collected on an FT-IR spectrometer (model FTIR-2000, Perkin Elmer, Dresden, Germany) by using KBr discs (thickness of 500 μm). The spectra were collected in the range from 4000 to 400 cm^−1^ at a resolution of 2 cm^−1^ (total of 20 co-added scans). The spectra presented were baseline corrected and converted into absorbance mode.

#### 2.5.7. Wide Angle X-ray Scattering

X-ray powder diffraction (XRD) patterns were recorded by using an XRD-diffractometer (Rigaku-Miniflex600, Chalgrove, Oxford, UK) with a Cu-Kα. The samples were scanned from 5° to 60° (step: 0.05 deg, scan speed: 1 deg/min).

#### 2.5.8. Differential Scanning Calorimetry (DSC)

Differential scanning calorimetry (DSC) studies were performed on a Pyris Diamond DSC system (Perkin–Elmer, Dresden, Germany). Indium and Zinc standards were used for system calibration. Accurately weighted samples (5 ± 0.1 mg) were sealed in aluminum pans and heated in the range between 30 and 320 °C at a heating rate of 20 °C/min.

#### 2.5.9. Measurement of Size and Surface Potential

Particle-size distribution and zeta potential were determined by dynamic light scattering (DLS) by using a Zetasizer Nano-S system (Malvern Instruments, UK). The self-optimization routine in the Zetasizer software (Version 3.30, Malvern Panalytical Ltd., Malvern, UK) was used for all measurements, and the zeta-potential was calculated according to the solutions theory. After a 100-fold dilution with a low ionic strength (2 mM) phosphate buffer at pH 7, measurements were performed at 25 °C in triplicate.

#### 2.5.10. Drug Loading, Yield and Entrapment Efficiency (EE)

For the determination of drug loading, 1 mg of the prepared nanoparticles was dissolved in dichloromethane:methanol in a 1:1 ratio, and the resultant solution was analyzed by HPLC (as described in Section 2.5.12. Drug loading, yield, and entrapment efficiency were calculated by using Equations (1)–(3), respectively:

(1) $$\mathrm{Drug}\;\mathrm{loading}\;\left(\%\right)=\frac{\mathrm{weight}\;\mathrm{of}\;\mathrm{drug}\;\mathrm{in}\;\mathrm{nanoparticles}}{\mathrm{weight}\;\mathrm{of}\;\mathrm{nanoparticles}}\times 100$$

(2) $$\mathrm{Yield}\;\left(\%\right)=\frac{\mathrm{weight}\;\mathrm{of}\;\mathrm{nanoparticles}}{\mathrm{initial}\;\mathrm{weight}\;\mathrm{of}\;\mathrm{polyesters}\;\mathrm{and}\;\mathrm{drug}}\times 100$$

(3) $$\mathrm{EE}\;\left(\%\right)=\frac{\mathrm{weight}\;\mathrm{of}\;\mathrm{drug}\;\mathrm{in}\;\mathrm{nanoparticles}}{\mathrm{initial}\;\mathrm{weight}\;\mathrm{of}\;\mathrm{drug}}\times 100$$

#### 2.5.11. In-Vitro Drug Release

For the in-vitro GAL release studies, aDistek Dissolution Apparatus (Evolution 2100C North Brunswick Township, NJ, USA) was used equipped with an autosampler (Evolution 4300, North Brunswick Township, NJ, USA). The method used was that of USP I (basket method). Nanoparticles of about 50 mg in weight were placed into suitable dialysis tubing cellulose membranes with Molecular Weight Cut-Off (MWCO) 12,000–14,000 (D9402-100FT, North Brunswick Township, NJ, USA) and inserted into the dissolution baskets, and the dissolution analysis was performed at 37 ± 1 °C at a rotation speed of 50 rpm. The dissolution medium was 500 mL of a phosphate buffered saline (PBS), pH = 7.4 solution. At predefined time intervals, 2 mL of aqueous solution was withdrawn from the release media and analyzed for galantamine content by HPLC, as described in Section 2.5.12. 

#### 2.5.12. HPLC Analysis

Galantamine quantification was determined by using a Shimadzu Prominence HPLC system that consisted of a degasser (Model DGU-20A5, Tokyo, Japan), a pump (Model LC-20AD, Tokyo, Japan), an autosampler (Model SIL-20AC, Tokyo, Japan), a UV–Vis detector (Model SPD-20A, Tokyo, Japan), and a column oven (Model CTO-20AC, Tokyo, Japan). HPLC analysis was performed with a C_18_ column (CNW Technologies Athena, 120 Å, 5 μm, 250 mm × 4.6 mm, Tokyo, Japan) at 25 °C. A 10 mM aqueous solution of KH_2_PO_4_ with a pH of 3.5 was used as phase A, and methanol was used as phase B. The mobile phase consisted of A/B 80/20 *v*/*v*. The flow rate was set at 1.0 mL/min, the injection volume was 10 μL, and the galantamine analysis was performed at 235 nm.

### 2.6. In Vivo Experiments

#### 2.6.1. Animals and Treatments

PLGA 65/35–HPC–GAL–Rhod nanoparticles were diluted in a 0.9% *w*/*v* sterile saline solution and intranasally delivered to healthy, adult, 4-month old, male Wistar rats. Each rat received 25 μL of the prepared solution in each nostril, as previously described (Hanafy et al., 2016, Bonaccorso et al., 2017). Briefly, rats were sedated with a ketamine (Imalgene; Merial S.A, France; 50 mg/kg) and xylazine (Sedaxylan; Eurovet Animal Health B.V., Handelsweg, The Netherlands; 5 mg/kg) mixture, and the tip of a 200 μL micropipette, containing the nanoparticles solution, was inserted in each nostril while rats were held in the upright position. Each rat received a GAL dose of 3 mg/kg. A control group of rats was treated with 25 μL of a 0.9% *w*/*v* sterile saline solution in each nostril. The clinical evaluations of all experimental animals and food consumption observations were performed at 24 and 48 h after IN administration in order to reveal any possible effects that were triggered by the administered nanoparticles. At 48 h after their IN administration, rats were anesthetized with the same ketamine/xylazine mixture and transcardially perfused with 100 mL of a normal saline 0.9% solution followed by 300 mL of a 4% paraformaldehyde (PFA) solution.

Experiments were carried out in the animal facility unit of the Laboratory of Anatomy, Histology and Embryology of the School of Veterinary Medicine of AUTh’s University department of Health Sciences (accreditation number EL-54-BIOexp-23) and received the approval of the Veterinary Directorate of the Prefecture of Central Macedonia (approval number 66629(261)/2 April 2019). All manipulations were in compliance with the revised European and national legislation on management of animals used for scientific purposes (European directive 2010/63/EE and Presidential Degree 56/2013).

#### 2.6.2. Tissue Preparation and Microscopic Evaluation

Fixed brains were dissected out, postfixed in a 4% PFA solution overnight, and embedded in paraffin. Embedded brains were cut all along their longitudinal axis, and serial coronal sections, 10 μm thick, were collected. Using the reference points that were established in the rat brain atlas of Paxinos and Watson [29], systematically selected serial coronal sections all along the rostro-caudal extend of the hippocampal formation (−1.40 to −6.80 posterior to bregma) were examined under a Nikon Eclipse C1 (EZ-C1 software, version 3.20; Nikon GmbH, Düsseldorf, Germany) confocal laser scanning microscope (CLSM) in Z- stack captured images (1μm optical thickness per plane). Our aim was to examine whether the IN-administered nanoparticles were able to reach the hippocampal formation and, if so, to study their dispersion in the hippocampal subregions.

## 3. Results and Discussion

### 3.1. Synthesis and Characterization of HPC

**Figure 1:**
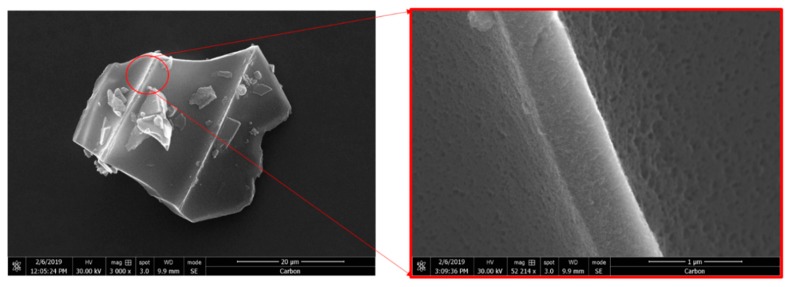
Representative SEM images of hierarchical porous carbon showing surface morphology and texture.

HPC was synthesized as previously described [25]. N_2_ adsorption experiments were performed to characterize the surface area and porosity. HPCs possess a high surface area (2200 m^2^/g) and pore volume, with the majority of the surface area arising from the mesopores size. Table 1 lists the textural characteristics of the HPC. SEM images (Figure 1) reveal the morphology of the HPC scaffold, with the macropores replicating the sublimated ice crystals.

X-ray photoelectron spectroscopy was used to characterize the chemical functionalities that were present on the HPC (Supplementary Materials Figure S1). The C–C peak was the dominant photoelectron peak at 285 eV, representing 71.8% of the whole carbon content. The peak that was centered at 286.2 eV was derived from the C–O bonds (11.8%), while the rest of the peaks at 287.3, 288.4, and 289.8 eV were due to C–O–C, C=O and C(O)O bonds representing 5.8%,3.5% and 3.7%, respectively, of the whole carbon content. Finally a weak photoelectron peak at 291.1 eV is due to the conjugated π-system of HPC.

**Figure 2:**
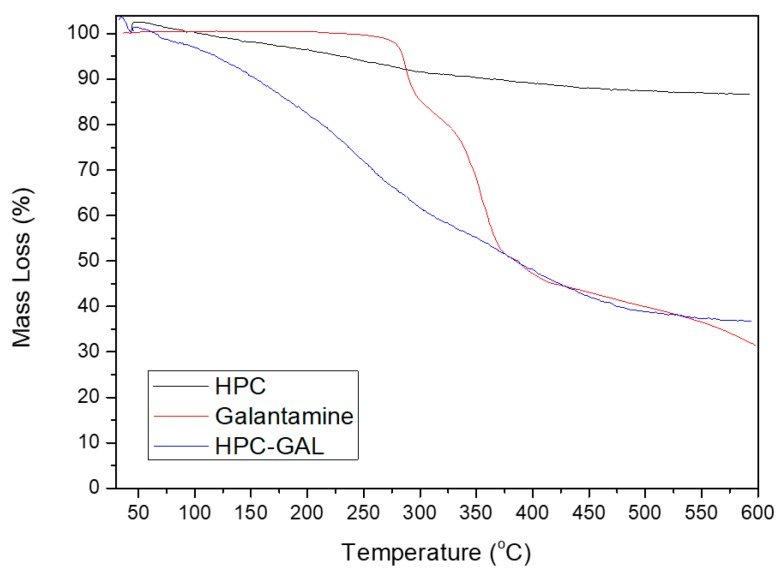
TGA curves of HPC and galantamine before and after its adsorption.

TGA was used in order to determine the quantity of the adsorbed galantamine. As can be observed in Figure 2, the HPC showed an initial mass loss of about 3% until 110 °C, probably due to the water humidity that was adsorbed from the atmosphere. Practically, HPC could be considered to be stable until that temperature. Thereafter and until 282.5 °C, there was an observed mass loss of about 7.5% and a second thermal degradation stage from 282.5 to 600 °C showing an additional mass loss of about 5%. In total, the mass loss was about 12.5%, high enough when considering that HPC is a form of carbon. We believe this amount was due to hydroxyl groups that were present on the HPC. The presence of hydroxyl groups can be crucial for hydrogen bond formation between HPC and galantamine after adsorption.

Galantamine thermally decomposed in three stages, with the first one starting at 268 °C and lasting at 297 °C, showing a corresponding mass loss of about 15%. The second stage started from 297 °C and lasteduntil around 373 °C, and it showed the highest mass loss (~32%). The third stage started from 373 °C and lasted to 600 °C, with an additional mass loss of about 23%. The total mass loss corresponded to 70%.

The TGA curve of the HPC–GAL showed an initial mass loss of about 1% until 64 °C and was probably due to the traces of methanol that remained after galantamine’s adsorbance from the methanol solution. Thereafter and until 268 °C, there was a mass loss of 29% due to HPC thermal decomposition because galantamine appeared to be stable until that temperature. The HPC–GAL showed a remaining mass loss of 37% at 600 °C; consequently, the mass loss between 268 and 600 °C was calculated to 31% (68% of the HPC–GAL at 268 °C minus 37% of the same sample at 600 °C). The mass loss of the HPC at that range (268 to 600 °C) was 9.5%. From the above, we conclude that 21.5% wt of the GAL was adsorbed on the HPC [22].

**Figure 3:**
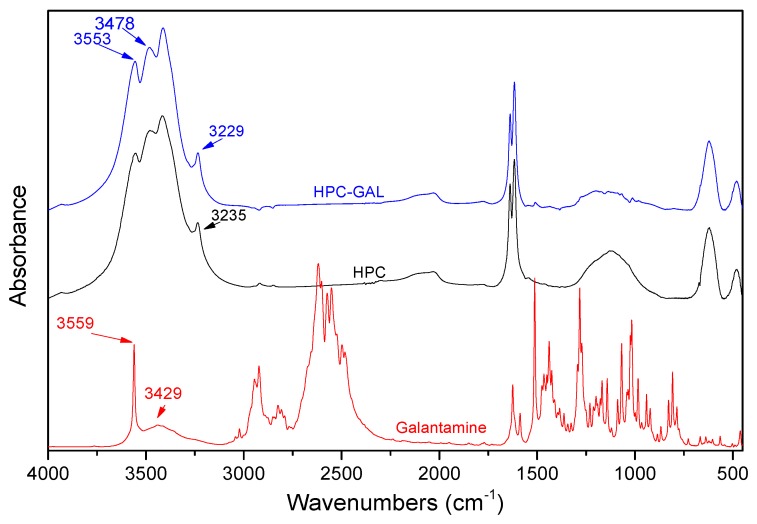
FT-IR spectra of the HPC, the galantamine, and of the mixture after adsorption.

Figure 3 shows the FT-IR spectra of the HPC, galantamine and HPC–GAL. The spectrum of galantamine showed peaks at 2834, 3429, and 3559 cm^−1^ due to the stretching vibration of the >C–H, >N–H and –OH bonds, respectively [30]. The FT-IR spectra of the HPC showed peaks at 1616 and 1639 cm^−1^ due to C=C bonds, at 3416, 3477 and 3553 cm^−1^ due to >C–H bonds, and finally a peak at 3235 cm^−1^ assigned to hydroxyl groups (–OH) on the surface of the HPC. The spectrum of the GAL/HPC contained peaks from both. A closer look at the spectrum revealed some shifting from the original peaks. The largest shift was for the >N–H stretching vibration of the galantamine from 3429 to 3478 cm^−1^, probably due to interactions between the amine group of the drug and the HPC.

**Figure 4:**
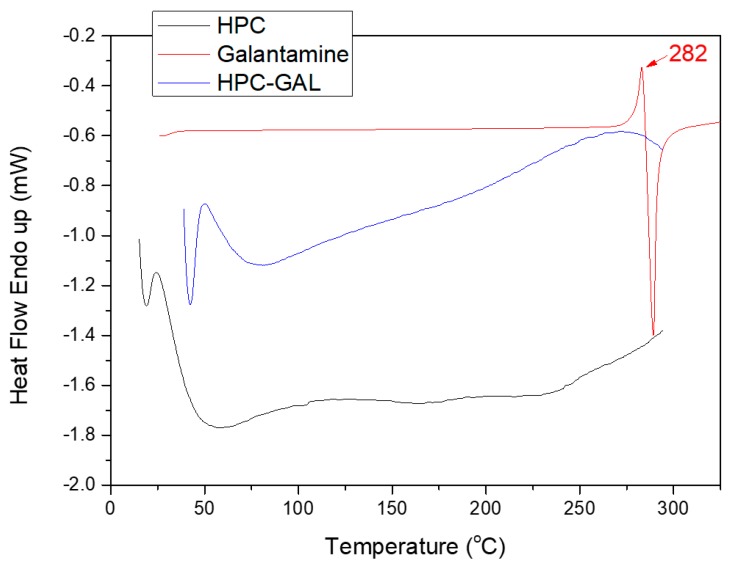
Differential scanning calorimetry (DSC) thermographs of HPC and galantamine before and after the latter’s adsorption.

X-ray diffraction analysis (Figure S2) was used to evaluate the crystallinity of the galantamine after its adsorption on the HPC. As can be seen, the neat galantamine was highly crystalline. The diffraction pattern of the neat drug showed two main, sharp peaks at 13.3 and 21.2 deg and many other lower intensity but fairly sharp peaks. Note that the HPC was amorphous with no diffraction peaks between 5 and 60 deg. After adsorption, the mixture showed no peaks, suggesting that the galantamine was amorphous when adsorbed on the HPC. These results were consistent with the DSC analysis (Figure 4), where the characteristic melting transition of the galantamine disappeared after it was adsorbed on the HPC.

DSC was also used in order to further study possible interactions between the drug and the HPC (Figure 4). The galantamine showed a characteristic endothermic peak at 282 °C, after which decomposition took place. This observation was in accordance with the TGA results. After its adsorption to the HPC, no melting peak of the galantamine was observed, which was consistent with the XRD data suggesting that the galantamine was present in its amorphous state.

### 3.2. Polymeric GAL Loaded Nanoparticles Characterization

**Figure 5:**
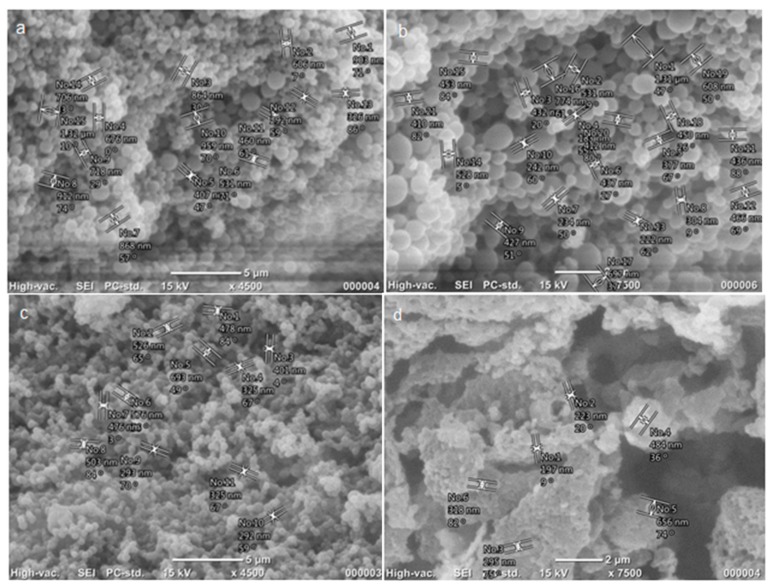
SEM micrographs of prepared nanoparticles (**a**) poly(l-lactic acid) (PLLA)–GAL, (**b**) poly(lactide-*co*-glycolide) (PLGA) 75/25–GAL, (**c**) PLGA 65/35–GAL, and (**d**) PLGA 65/35–HPC–GAL.

SEM imaging was used to characterize the size and morphology of the PLGA nanoparticles after the encapsulation of the GAL- and HPC–GAL-loaded particles. As seen in Figure 5, the nanoparticles were spherical in shape with a smooth surface and no agglomeration.

The average size of the various nanoparticles systems and their zeta potential were also measured, and the results are summarized in Table 2. The size of neat nanoparticles varied between 134 and 149 nm and showed low polydispersity index (PDI) values. The size of the nanoparticles with encapsulated galantamine increased compared with the neat ones. Finally, the size of the nanoparticles that were prepared with HPC–GALfurther increased. The PLLA-based systems showed the highest increases, with sizes of 1300 nm. In contrast, the size of the PLGA-based nanoparticles were 394 and 242 nm for the 75/25 and 65/35 ratios, respectively. For all prepared nanoparticles, the zeta potential was negative and varied between −17 and −29 mV.

**Figure 6:**
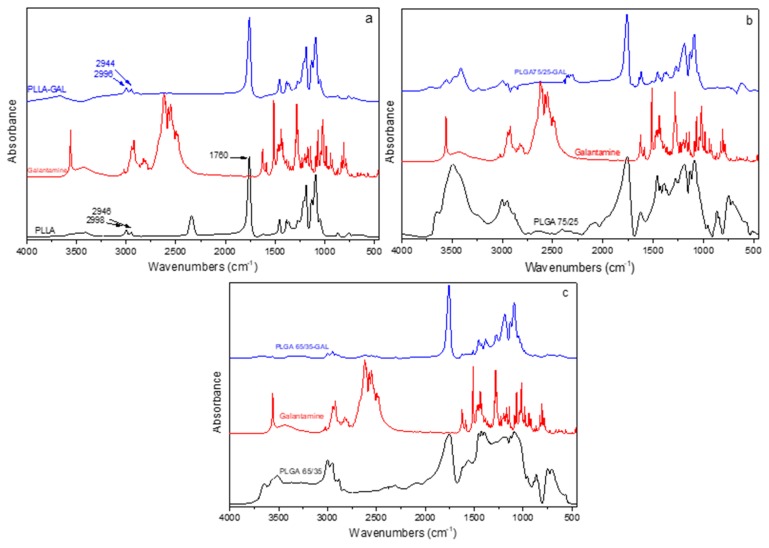
FT-IR spectra of neat polymers and their nanoparticles with galantamine. (**a**) PLLA–GAL, (**b**) PLGA 75/25–GAL, and (**c**) PLGA 65/35–GAL.

FT-IR was used to study possible interactions between the polymers and the galantamine or the HPC–GAL. Figure 6a shows the FT-IR spectra of the neat PLLA, galantamine and nanoparticles that were formed. As can be observed, PLLA showed its characteristic peaks, a double peak at 2998 and 2946 cm^−1^ due to the stretching vibrations of the >C–H bond and a peak at 1760 cm^−1^ due to the stretching vibrations of the >C=O bonds [22]. The spectra of the PLLA–GAL nanoparticles showed peaks at 2622, 1512, 1426, and 1384 cm^−1^ due to the galantamine (neat galantamine showed peaks at 2620, 1514, 1425 and 1385 cm^−1^). The FT-IR spectra of the PLGA (both 75/25 and 65/35 *w*/*w*) are shown in Figure 6b,c; a double peak at 2997 and 2952 cm^−1^ was due to the stretching vibration of >C–H, a peak at 1757 cm^−1^ was due to the stretching vibrations of the >C=O bonds and the aliphatic ether C–O–C stretch at 1088 cm^−1^ [22]. Small shifts from the original peaks in the neat materials to lower wavenumbers were observed for the galantamine-containing nanoparticles.

**Figure 7:**
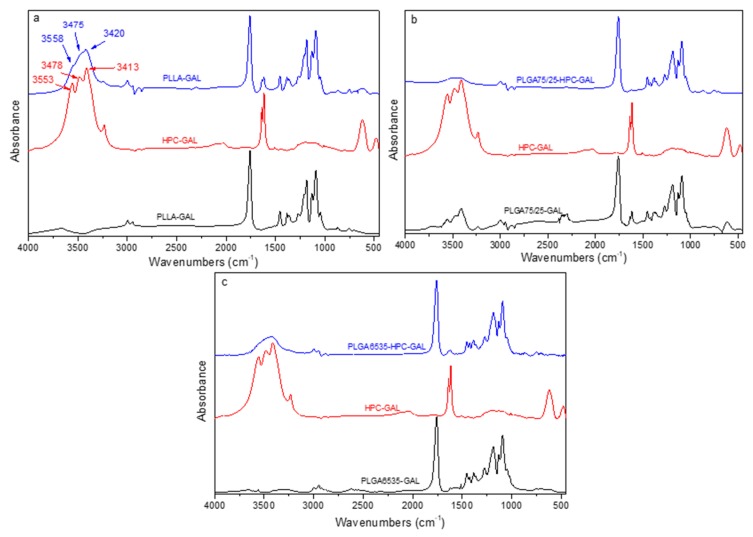
FT-IR spectra of nanoparticles with HPC–GAL. (**a**) PLLA–HPC–GAL, (**b**) PLGA 75/25–HPC–GAL, and (**c**) PLGA 65/35–HPC–GAL.

Figure 7a–c shows the FT-IR spectra of the nanoparticles that contained HPC–GAL compared to HPC–GAL and nanoparticles with galantamine alone. No significant changes were observed in the region 3000–450 cm^−1^. A more significant change in the spectra of the nanoparticles containing the HPC–GAL was observed in the region 3800–3000 cm^−1^, where there were chemical shifts from 3413, 3478, and 3553 cm^−1^ of the HPC–GAL to 3420, 3475 and 3558 cm^−1^ for the PLLA nanoparticles (Figure 7a). The results with respect to PLGA nanoparticles in both ratios were analogous (Figure 7b,c). These small shifts indicated that interactions took place between the drug and the polymer, possible due to hydrogen bond formation.

**Figure 8:**
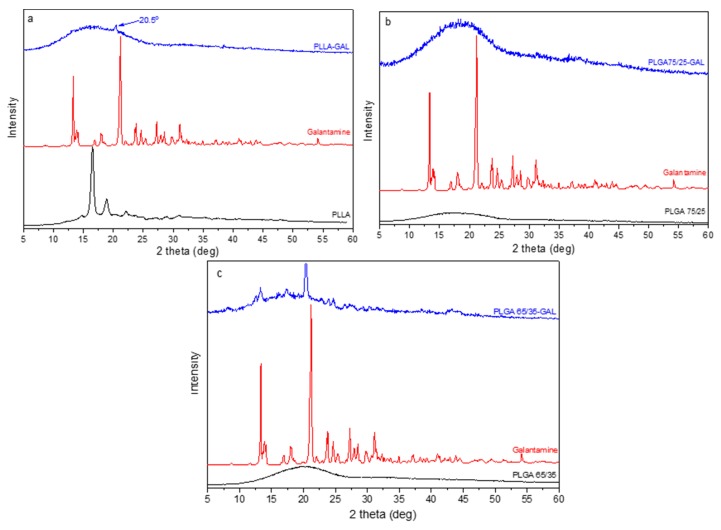
XRD patterns of neat polymers and their nanoparticles with galantamine. (**a**) PLLA–GAL, (**b**) PLGA 75/25–GAL, and (**c**) PLGA 65/35–GAL.

The prepared nanoparticles were also studied for their crystallinity by XRD. Figure 8 shows the diffractograms that were obtained for neat polymers and their nanoparticles with galantamine. PLLA (Figure 8a) showed two characteristic main peaks at 16.5 and 18.8 deg and two smaller ones at 14.7 and 22.2 deg, indicating that it is a semicrystalline polymer [22], while PLGA copolymers are completely amorphous. In both XRD patterns of these copolymers, a very broad peak was recorded (Figure 8b,c). In the GAL-loaded nanoparticles, all the peaks due to either PLLA or to the drug were absent, and a small new peak appeared at 20.5 deg, indicating a small amount of crystallinity in the nanoparticles. The new peak could be attributed to the different crystal form of galantamine. The same also appeared in nanoparticles of PLGA 65/35 (Figure 8c) because some lower intensity peaks close to the galantamine’s peaks were recorded. This is an indication that the drug was present in its crystalline state in the nanoparticles but in a state that was different than the neat drug. For the 75/25 *w*/*w* ratio (Figure 8b), no crystalline peaks were observed in the nanoparticles, probably due to the low quantity of the drug that was entrapped or due to the complete amorphization of the drug.

**Figure 9:**
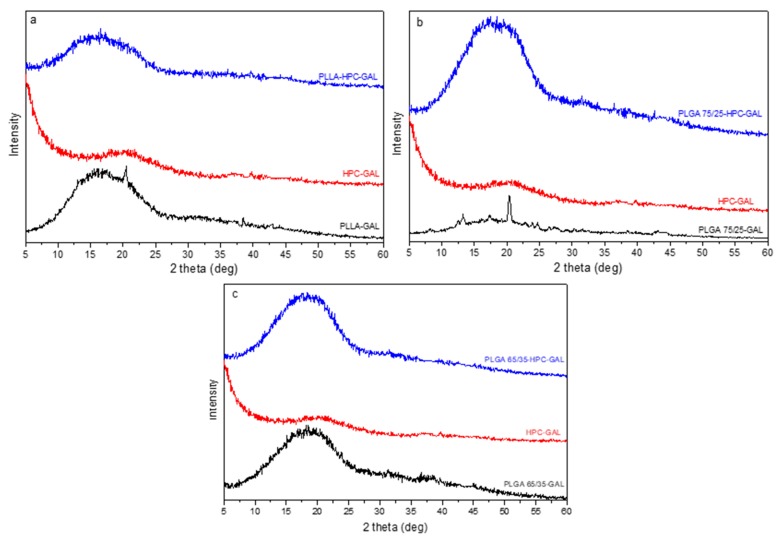
XRD patterns of nanoparticles with HPC–GAL. (**a**) PLLA–HPC–GAL, (**b**) PLGA 75/25–HPC–GAL, and (**c**) PLGA 65/35–HPC–GAL.

For nanoparticles that were prepared with HPC–GAL (Figure 9) no peak was seen, indicating that the formulations were amorphous. This was expected, since, as was mentioned previously, GAL loaded in HPC enhanced its amorphization.

**Figure 10:**
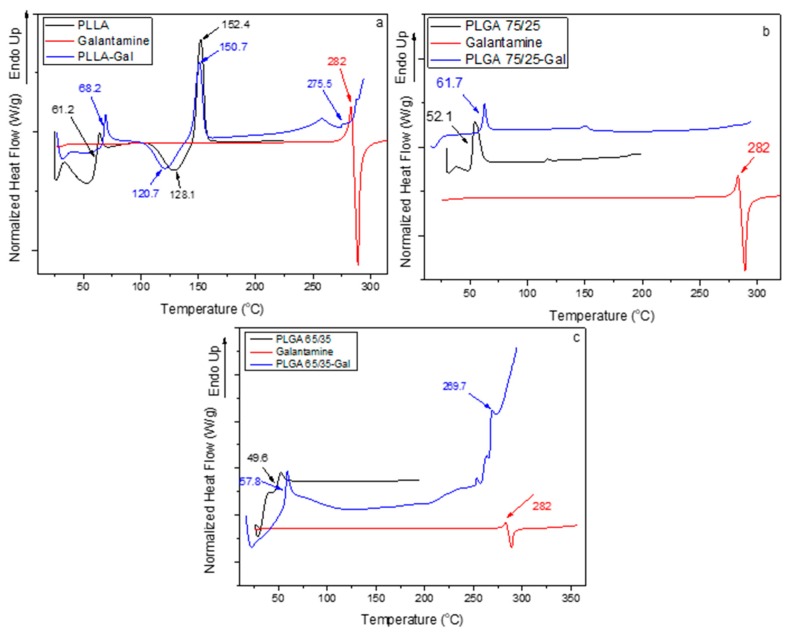
DSC thermographs of neat polymers and their nanoparticles with galantamine (**a**) PLLA–GAL, (**b**) PLGA 75/25–GAL, and (**c**) PLGA 65/35–GAL.

DSC was further used in order to verify the crystallinity of the galantamine in the nanoparticles. As can be observed, PLLA showed a glass transition temperature (T_g_) at 61.2 °C, a cold crystallization temperature (T_cc_) at 128.1 °C, and a melting temperature (T_m_) at 152.4 °C [22]. The incorporation of galantamine affected the structure of nanoparticles. As can be observed, the characteristic values for PLLA changed to 68.2, 120.7, and 150.7 °C, suggesting that interactions took place between the drug and the polymer. Furthermore, a new peak at 275.5 °C appeared in the thermograph due to the encapsulated drug, further confirming the XRD results (Figure 10a) that galantamine was present in its crystalline structure in the nanoparticles. The shift of T_m_ suggested that the galantamine’s structure was slightly affected by the polymer. Moreover, the reduction of T_cc_ of the neat PLLA in the nanoparticles showed that the incorporation of the drug facilitated cold crystallization, probably due to the formation of less entangled polymer chains.

The results for PLGA nanoparticles were analogous (Figure 10b,c). PLGA with the 75/25 and 65/35 ratios showed T_g_ points of 52.1 and 49.6 °C, respectively, that were shifted to higher values, i.e., at 61.7 and 57.8 °C after nanoparticle formation. No T_cc_ and T_m_ points were observed for PLGA, since it is amorphous. The T_m_ of galantamine was decreased to 280.1 and 269.7 °C, indicating that the drug was present in its crystalline form and slightly affected after the formation of the nanoparticles.

As was shown by DSC (Figure 4), the galantamine became amorphous after its adsorption to HPC. It was expected that in the nanoparticles, the drug would also be amorphous, something that was confirmed by the XRD (Figure 9) and DSC results (Figure S3). In all thermographs, the characteristic peaks of the polymer were present, but the T_m_ of the drug was not, further supporting that HPC–GAL remained amorphous after its incorporation in the polymeric nanoparticles.

### 3.3. Dissolution Study

Table 3 contains the yield, entrapment efficiency, and drug loading for various nanoparticle systems.

The drug loading of the neat galantamine showed values ranging between 5.34% and 9.57%. These results are consistent with the fact that galantamine is water soluble and that a significant amount is expected to be transferred to the aqueous phase during nanoparticle preparation. When the drug was incorporated into the HPC, the drug loading increased substantially until about 30%, suggesting that the HPC sequestered and protected the galantamine during nanoencapsulation. These nanoparticles also showed the highest values of drug loading and entrapment efficiency.

**Figure 11:**
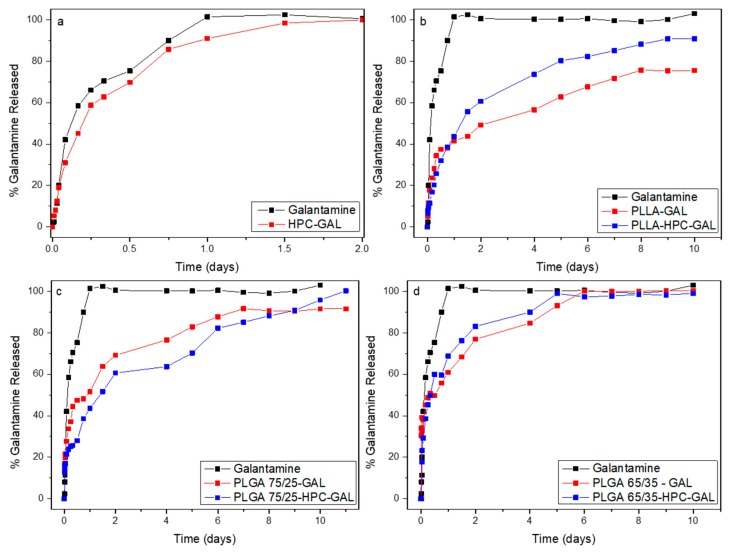
In vitro galantamine release profile. (**a**) Gal and HPC-GAL; (**b**)PLLA nanoparticles; (**c**) PLGA 75/25 nanoparticles; (**d**) PLGA 65/35 nanoparticles.

Figure 11 shows the dissolution profile in vitro. The release of galantamine from HPC reached 100% after two days. The release profile showedfour stages; the first stage was complete after 45 min with 20% of the drug released. The second stage took 6 h, with a release reaching about 60%. The third stage lasted 18 h, after which 85% release was achieved. Finally, 100% release was achieved after two days, which was consistent with previous results. Note that the dissolution profile of the neat galantamine showed a similar behavior except that the amounts were higher at similar times.

The direct incorporation of GAL into PLLA nanoparticles led to a different release profile. In that system, 37% of the galantamine was released in the first 12 h, with the release reaching a steady state of about 75% in nine days and not much change afterwards. Nanoparticles containing GAL/HPC showed a sustained release mode; in the first stage, which lasted 1.5 days, the release reached 55%, and a second stage that lasted for nine days reached about 90% of release. It seemed that PLLA led the galantamine to be released in a controlled manner, while the HPC was further enhanced its dissolution, probably due to its protection to the drug.

A completely different dissolution profile could be seen in the nanoparticles that were prepared by PLGA 75/25 (Figure 11c). Concerning the nanoparticles with the neat galantamine, it was observed that in the first 18 h, the release reached at about 50%, and, until 24 h, no further drug was released. Thereafter and until two days, there was a release of about 20%, followed by reduced rate resulting in 70%, and a final linear release of 90% in seven days. When the drug was adsorbed in the HPC, small changes to the profile were observed, i.e., a first stop in the dissolution of the galantamine was observed between 12 and 18 h and a second one was observed between two and four days, after which no significant changes were observed. The linear dissolution profile was also observed beginning at six days and lastinguntil 11 days, reaching about a 100% release of the drug.

**Figure 12:**
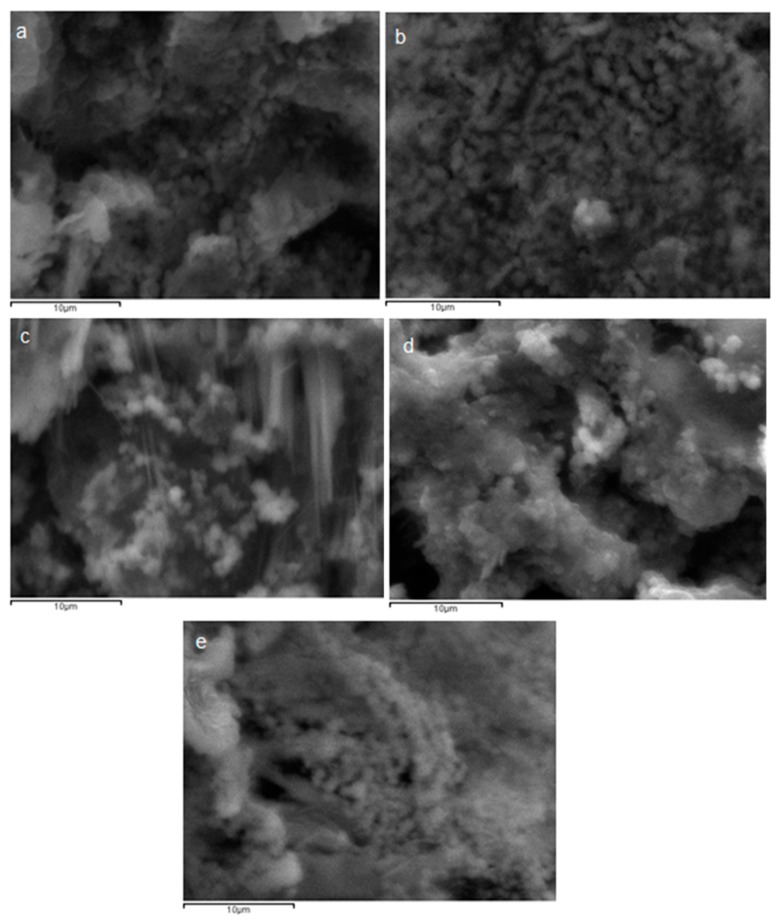
SEM photos of nanoparticles after dissolution study. (**a**) PLLA–GAL, (**b**) PLGA 75/25–GAL, (**c**) PLGA 65/35–GAL, (**d**) PLGA 75/25–HPC–GAL, and (**e**) PLGA 65/35–HPC–GAL.

There were small changes that were observed depending on the nature of the polymer. We attributed these differences to differences in the size and dissolution of the nanoparticles due their different chemical natures. An examination of the nanoparticles after dissolution showed that the PLA nanoparticles retained their spherical morphology and size, suggesting that the drug release took place by diffusion only (Figure 12). In contrast, both PLGA nanoparticle systems appeared featureless after dissolution, suggesting that the dissolution of the nanoparticles also took place in addition to the diffusion of the drug.

### 3.4. In Vivo Experimentation

#### 3.4.1. Selection and Fluorescence Evaluation of Rhodamine-Treated PLGA 65/35–HPC–GAL Nanoparticles for IN Administration in Rats

PLGA nanoparticles are widely used for the nose-to-brain delivery of various pharmaceutical substances because of their high mucoadhesive properties [31]. In the present study, PLGA 65/35-HPC–GAL nanoparticles were selected for in vivo administration due to their relative smaller size and lower polydispersity when compared to the PLGA 75/25–HPC–GAL and PLLA–HPC–GAL nanoparticles. Moreover, PLGA nanoparticles were shown to display an increased uptake by the olfactory unsheathing cells when compared to PLA nanoparticles [32]. PLGA 65/35–HPC–GAL nanoparticles showed an average size of 240 nm (Table 2), and existing literature suggests that PLGA nanoparticles of an average size of <300 nm are capable of entering the brain just a few hours after their IN administration in adult Wistar rats [33]. Taking into account all the above, the PLGA 65/35–HPC–GAL nanoparticles were expected to be more effectively delivered to the brain by comparison to the significantly larger PLGA 75/25–HPC–GAL (393.959 ± 0.39 nm) and PLLA–HPC–GAL nanoparticles (1302.06 ± 0.49 nm).

Rhodamine B was successfully encapsulated in the PLGA 65/35–HPC–GAL nanoparticles in order to allow for their fluorescent detection in the brain parenchyma. Before their IN administration, PLGA 65/35–HPC–GAL–Rhod nanoparticles were scanned under the CLSM to confirm that they produced a strong red fluorescence signal that would allow for their intra-brain detection. The Rhod-treated PLGA 65/35–HPC–GAL nanoparticles showed a strong red fluorescence signal in the CLSM-captured images. No CLSM signal was observed when scanning the sole preparations of the HPC, PLGA 65/35, or galantamine. Our findings were further evaluated with spectroscopy.

**Figure 13:**
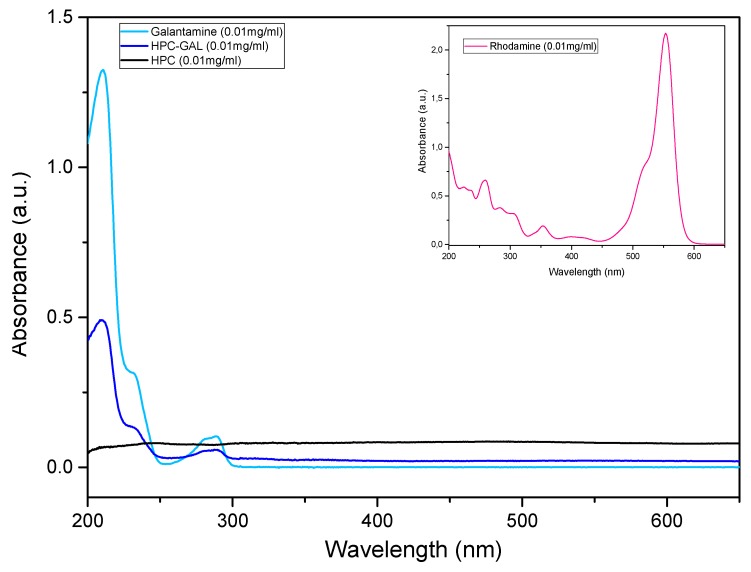
UV–Vis absorption spectra for water solutions of Rhodamine, galantamine, HPC and HPC–GAL.

The UV–Vis absorption spectra for water solutions of Rhod, GAL, HPC, and HPC–GAL at the same concentration (0.01 mg/mL) are shown in Figure 13. HPC was inactive in UV–Vis radiation, as long as no functional group exists. In contrast, a mean weak peak appeared at 210 nm in the HPC–GAL due to the GAL’s immobilization into the porous structure of the HPC. Additionally, Rhod displayed only an intense peak at 554 nm.

#### 3.4.2. PLGA 65/35–HPC–GAL Nanoparticles in the Hippocampus of the Rat Brain Following IN Administration

The hippocampus, which is responsible for memory formation and navigation, is a brain region that is primarily and severely degenerated in AD, leading to the onset of clinical manifestations [34]. Galantamine hydrobromide is a known competitive inhibitor of AChE that also increases receptors’ sensitivity to acetylcholine (Ach), enabling, in this way, synaptic transmission in AD-suffering hippocampus [35]. At 48 h after their IN administration, PLGA 65/35-HPC–GAL–Rhod nanoparticles were detected in the hippocampus of all experimental rats. This was in full accordance with previous studies demonstrating that the uptake of PLGA nanoparticles in the rat brain is significantly increased 48 h after their IN administration [11]. No fluorescence was detected in the hippocampi of the IN saline-treated rats.

**Figure 14:**
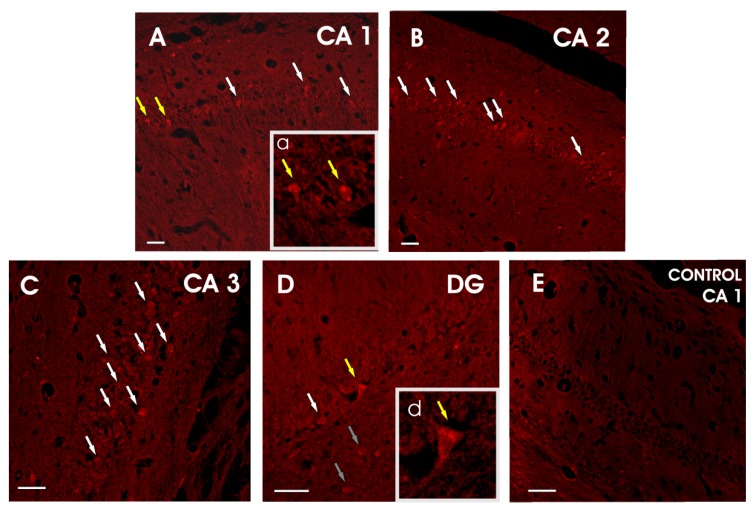
Photomicrographs illustrating the uptake of PLGA 65/35-HPC–GAL–Rhod nanoparticles by neurons of all layers of the entire hippocampal formation at 48 h after their intranasal (IN) delivery. Administered nanoparticles were found intra-neuronally in the pyramidal neurons of the CA1 (white and yellow arrows) (**A**), CA2 (white arrows) (**B**), and CA3 (white arrows) (**C**) fields and also in the mossy cells (grey arrows) (**D**) and granule cells of the DG (white and yellow arrows) (**D**). PLGA 65/35–HPC–GAL–Rhod nanoparticles formed aggregates that were distributed in the cytoplasm of hippocampal neurons (yellow arrows, a and d). No fluorescence was seen in hippocampal sections of the IN saline-treated rats (**E**). Scale bar = 50 μm.

Administered nanoparticles were found in all layers of the entire hippocampal formation, namely the Cornu Ammonis (CA) region 1 (CA1), 2 (CA2), 3 (CA3), 4 (CA4 or hilus), and dentate gyrus (DG) (Figure 14). The distribution of nanoparticles was prevalent in the CA1, 2 and 3 regions, while only few granule cells were found to have incorporated administered nanoparticles in the DG.

In all hippocampal layers the IN delivered nanoparticles were located intra-neuronally, and no nanoparticles were found in the peri-neuronal space. More specifically, nanoparticle accumulations were detected in the cytoplasm of both pyramidal neurons (primary neurons of the CA fields) and granule cells (primary neurons of the DG). In a previous study by Bonaccorso et al. [11], chitosan/PLGA nanoparticles were also detected in the cytoplasm of hippocampal neurons after their IN administration. Shang and colleagues [36] reported that not only the polymeric properties but also the size of polymeric nanoparticles determine their uptake efficiency and internalization when reacting with living cells, proposing that nanoparticles with an average size of 200–500 nm are capable to enter the cells via endocytosis, mostly involving a caveolae-mediated mechanism [37].

The potential safety of the one dose, IN-delivered PLGA 65/35–HPC–GAL–Rhod nanoparticles was also evaluated. All experimental rats were clinically examined on a daily basis and inspected for possible signs of toxicity. The health status of all animals was found to be excellent, and no findings indicating possible toxicity occurred. The food consumption of experimental animals was also examined, and no decrease in food intake was observed all through the 48 h period after IN administration. Administered nanoparticles had an average size of 240 nm, and nanoparticles of various synthesis with sizes >100 nm were shown to have no cytotoxic effects on incorporating neurons, whereas the incorporation of smaller nanoparticles favored intra-neuronal adverse chemical reactions, such as increased reactive oxygen species (ROS) generation and disturbed calcium homeostasis [38,39,40]. However, the potential safety of IN-administered PLGA 65/35–HPC–GAL nanoparticles has to be further examined after repeated IN administrations.

## 4. Conclusions

Hybrid nanoparticles based on GAL/HPC encapsulated into PLA and PLGA were developed. The incorporation of GAL into HPC led to an amorphous form thatpositively influenced the dissolution and release profile of the drug. In vivo studies showed that IN-administered GAL that was encapsulated in PLGA-coated carbon nanoforms was successfully delivered to the hippocampus just a few hours after a single IN dose and shed light into the nanoparticle’s distribution in the distinct structural and functional hippocampal compartments that are differentially implicated in AD onset and progress. However, the potential safety and benefits from schemes of IN-administered PLGA–HPC–GAL nanoparticles in rodent models of AD have yet to be evaluated.

## Figures and Tables

**Table 1:** Textural characteristics of hierarchical porous carbon (HPC).

	BET Surface Area [m^2^ g^−1^]	*t*-Plot Micropore [m^2^ g^−1^]	Mesopore Surface [m^2^ g^−1^]	Total Pore Volume [cm^3^ g^−1^]	Mesopore Volume [cm^3^ g^−1^]	Micropore Volume [cm^3^ g^−1^]	Vmeso/Vtotal
HPC	2211	233	1978	4.014	3.90	0.112	97

**Table 2:** Average size and ζ-potential for various nanoparticle systems that were obtained by dynamic light scattering (DLS).

Sample (Nanoparticles)	Average Size (nm)	PDI	Z-Potential
neat PLLA	149.00 ± 0.01	0.12 ± 0.04	−20.37 ± 0.02
neat PLGA 75/25	138.00 ± 0.01	0.17 ± 0.05	−25.50 ± 0.03
neat PLGA 65/35	134.00 ± 0.02	0.14 ± 0.03	−28.50 ± 0.03
PLLA–GAL	182.67 ± 0.01	0.21 ± 0.07	−29.37 ± 0.02
PLGA 75/25–GAL	224.00 ± 0.02	0.27 ± 0.05	−17.00 ± 0.08
PLGA 65/35–GAL	198.00 ± 0.02	0.25 ± 0.04	−27.42 ± 0.03
PLLA–HPC–GAL	1302.06 ± 0.49	0.97 ± 0.06	−20.27 ± 0.04
PLGA 75/25–HPC–GAL	393.96 ± 0.39	0.94 ± 0.12	−19.13 ± 0.12
PLGA 65/35–HPC–GAL	241.60± 0.31	1.00 ± 0.01	−19.77 ± 0.08

**Table 3:** Drug loading (%), entrapment efficiency (%), and nanoparticle yield (%).

Nanoparticles	Drug Loading (%)	Entrapment Efficiency (%)	Nanoparticle Yield (%)
PLLA–GAL	5.34 ± 0.24	25.14 ± 2.57	87.26 ± 2.05
PLGA 75/25–GAL	8.49 ± 0.72	28.49 ± 1.08	92.86 ± 1.98
PLGA 65/35–GAL	9.57 ± 0.61	29.04 ± 2.19	89.71 ± 2.14
PLLA–HPC–GAL	28.35 ± 1.06	54.04 ± 2.46	82.18 ± 1.57
PLGA 75/25–HPC–GAL	32.83 ± 1.84	59.23 ± 2.75	88.83 ± 2.34
PLGA 65/35–HPC–GAL	31.24 ± 1.75	58.76 ± 3.51	90.29 ± 3.08

## Supplementary Materials

The following are available online at https://www.mdpi.com/1999-4923/12/3/227/s1, Figure S1: Carbon 1s photoelectron peak of HPC, Figure S2: XRD spectra of HPC and galantamine before and after its adsorption, Figure S3: DSC thermoghraphs of nanoparticles with HPC-GAL (a) PLLA-HPC-GAL, (b) PLGA 75/25-HPC-GAL, (c) PLGA 65/35-HPC-GAL.
