# Supplementary material for: Hierarchical Porous Carbon—PLLA and PLGA Hybrid Nanoparticles for Intranasal Delivery of Galantamine for Alzheimer’s Disease Therapy
Source: Pharmaceutics. 2020 Mar 4;12(3):227. doi: 10.3390/pharmaceutics12030227 (PMC7150929; doi:10.3390/pharmaceutics12030227)
Supplement: Supplementary file 1 [file pharmaceutics-12-00227-s001.pdf]

pharmaceutics

Supplementary Materials: Hierarchical Porous Carbon—PLLA and PLGA Hybrid Nanoparticles for Intranasal Delivery of Galantamine for Alzheimer's Disease Therapy

Stavroula G. Nanaki, Konstantinos Spyrou, Chryssa Bekiari, Pelagia Veneti, Turki N. Baroud, Niki Karouta, Ioannis Grivas, Georgios C. Papadopoulos, Dimitrios Gournis and Dimitrios N. Bikiaris

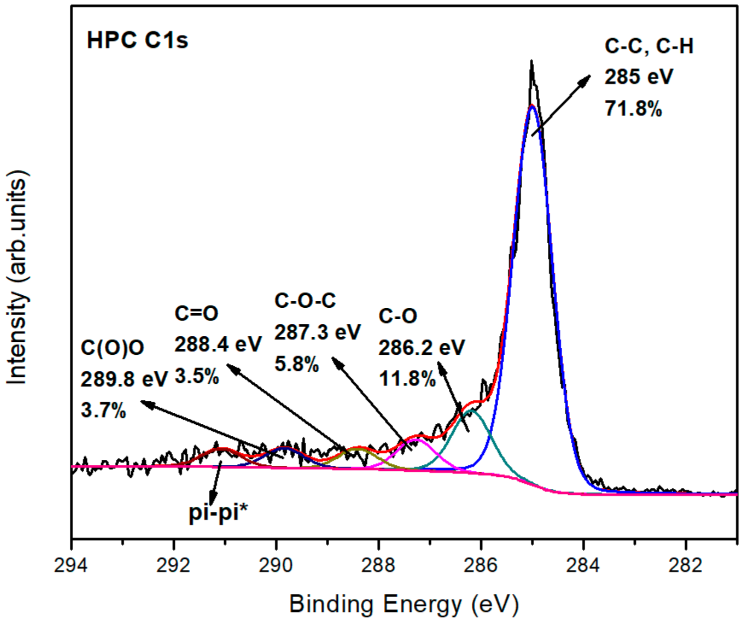

Figure S1. Carbon 1s photoelectron peak of HPC.

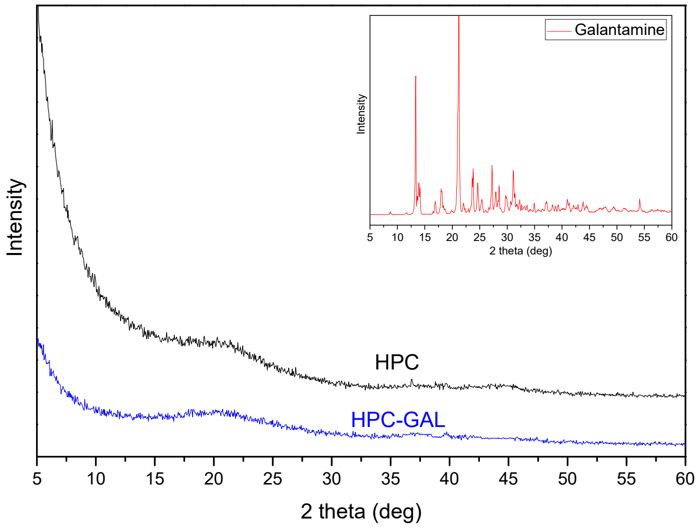

Figure S2. XRD spectra of HPC and galantamine before and after its adsorption.

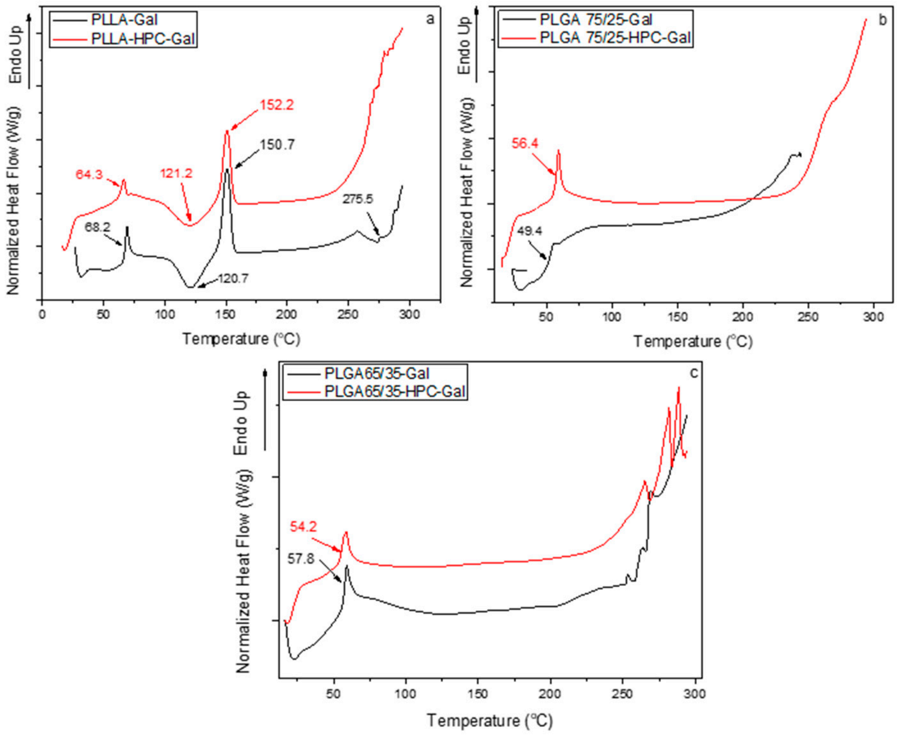

Figure S3. DSC thermoghraphs of nanoparticles with HPC-GAL (**a**) PLLA-HPC-GAL, (**b**) PLGA 75/25-HPC-GAL, (**c**) PLGA 65/35-HPC-GAL.
